# First-Year Evaluation of Mexico’s Tax on Nonessential Energy-Dense Foods: An Observational Study

**DOI:** 10.1371/journal.pmed.1002057

**Published:** 2016-07-05

**Authors:** Carolina Batis, Juan A. Rivera, Barry M. Popkin, Lindsey Smith Taillie

**Affiliations:** 1 Center for Nutrition and Health Research, National Institute of Public Health, Cuernavaca, Morelos, Mexico; 2 National Council for Science and Technology CONACYT, Mexico City, Mexico; 3 Department of Nutrition, Gillings School of Global Public Health, Carolina Population Center, University of North Carolina, Chapel Hill, Chapel Hill, North Carolina, United States of America; University of Cambridge, UNITED KINGDOM

## Abstract

**Background:**

In an effort to prevent continued increases in obesity and diabetes, in January 2014, the Mexican government implemented an 8% tax on nonessential foods with energy density ≥275 kcal/100 g and a peso-per-liter tax on sugar-sweetened beverages (SSBs). Limited rigorous evaluations of food taxes exist worldwide. The objective of this study was to examine changes in volume of taxed and untaxed packaged food purchases in response to these taxes in the entire sample and stratified by socioeconomic status (SES).

**Methods and Findings:**

This study uses data on household packaged food purchases representative of the Mexican urban population from The Nielsen Company’s Mexico Consumer Panel Services (CPS). We included 6,248 households that participated in the Nielsen CPS in at least 2 mo during 2012–2014; average household follow-up was 32.7 mo. We analyzed the volume of purchases of taxed and untaxed foods from January 2012 to December 2014, using a longitudinal, fixed-effects model that adjusted for preexisting trends to test whether the observed post-tax trend was significantly different from the one expected based on the pre-tax trend. We controlled for household characteristics and contextual factors like minimum salary and unemployment rate. The mean volume of purchases of taxed foods in 2014 changed by -25 g (95% confidence interval = -46, -11) per capita per month, or a 5.1% change beyond what would have been expected based on pre-tax (2012–2013) trends, with no corresponding change in purchases of untaxed foods. Low SES households purchased on average 10.2% less taxed foods than expected (-44 [–72, –16] g per capita per month); medium SES households purchased 5.8% less taxed foods than expected (-28 [–46, –11] g per capita per month), whereas high SES households’ purchases did not change. The main limitations of our findings are the inability to infer causality because the taxes were implemented at the national level (lack of control group), our sample is only representative of urban areas, we only have 2 y of data prior to the tax, and, as with any consumer panel survey, we did not capture all foods purchased by the household.

**Conclusions:**

Household purchases of nonessential energy-dense foods declined in the first year after the implementation of Mexico’s SSB and nonessential foods taxes. Future studies should evaluate the impact of the taxes on overall energy intake, dietary quality, and food purchase patterns (see S1 Abstract in Spanish).

## Abstract 2

An 8% tax on high-energy, non-essential foods in Mexico reduces the amount of these foods consumed, an effect that is more pronounced in lower income families, Taillie and colleagues reveal.

## Author Summary

- **Why Was This Study Done?**
  - In January 2014, Mexico passed an 8% tax on nonessential foods with energy density ≥275 kcal/100 g, including salty snacks, chips, cakes, pastries, and frozen desserts; and a 1 peso/liter (~10%) tax on sugar-sweetened beverages.
  - To date, there has been very limited research as to how larger health-related food/beverage taxes change household food purchases, or whether low socioeconomic status (SES) households are more responsive to such taxes.
- **What Did the Researchers Do and Find?**
  - Using a dataset that follows household food purchases over time, we examined whether the volume of taxed foods showed greater declines in the post-tax period than we would have expected based on trends in the volume of taxed food purchases prior to the tax. We also examined whether post-tax changes in the volume of taxed food purchases was greater among low SES households.
  - We found that the mean volume of purchases of taxed foods in 2014 declined by 25 g per capita per month, or a 5.1% change beyond what would have been expected based on pre-tax (2012–2013) trends.
  - There were no changes in the purchase of untaxed foods in the post-tax period.
  - Low SES households’ purchases of taxed foods declined by 10.2% and medium SES households by 5.8%, whereas high SES did not change.
- **What Do These Findings Mean?**
  - These findings show that in the post-tax period, purchases of taxed foods declined more than we would expect if pre-tax trends had simply continued, particularly among low and medium SES households. Future research should explore how these shifts are linked to changes in the nutritional quality of the overall diet.

## Introduction

Currently, the prevalence of overweight and obesity in Mexico is over 33% for children and about 70% for adults [1,2], and, in 2006, the prevalence of type 2 diabetes in adults was 14.4% [3]. Concurrent with the rise in obesity and diabetes were large increases in sugar-sweetened beverage (SSB) and nonessential energy-dense food (often termed “junk food”) intake [4–7]. Worldwide, Mexico is the fourth largest per-capita consumer of energy-dense, ultraprocessed food and drinks, including SSBs, sweet and savory snacks, breakfast cereals, confectionery, ice cream, biscuits, spreads, sauces, and ready-meals [8]. To prevent continued increases in obesity and diabetes, in January 2014, the Mexican government implemented a 1 peso-per-liter tax on SSBs (equivalent to approximately 10% tax) and an 8% tax on nonessential foods with energy density ≥275 kcal/100 g. In Mexico, total prices including the tax price are included on the shelf label, so the price consumers see includes the tax. The law defined nonessential foods in the following categories: chips and snacks, candies and sweets, chocolate, puddings, peanut and hazelnut butters, ice cream and ice pops, and cereal-based products with substantial added sugar. Based on the 2012 National Health and Nutrition Survey (ENSANUT), the intake of non-basic foods high in sugar or fat (a food classification similar to the tax) contributes 11% to 18% of daily caloric intake across age groups [9].

Worldwide, there is very limited empirical evidence on the effect of food/nutrient taxes [10,11]. While analysis of Mexican food and beverage taxes revealed that during 2014 purchases of taxed beverages declined 6% beyond what was expected compared to pre-existing trends, it is unclear whether taxed food purchases also declined, or whether households of lower socioeconomic status (SES) were more or less responsive.

Because both the nonessential energy-dense food and the SSB taxes were implemented concurrently, we cannot evaluate the independent effect of each. Therefore, the objective of the current work was to longitudinally examine changes in the volume of taxed and untaxed food purchases after both taxes were implemented, relative to the counterfactual (i.e., expected volume of taxed and untaxed food purchases if the taxes had not been implemented), overall and by SES subgroups.

## Methods

### Participants

This study uses data on volume of household food purchases from January 2012 to December 2014 from The Nielsen Company’s Mexico Consumer Panel Services (CPS). The analysis used de-identified data and was granted an exemption from the University of Chapel Hill and National Institute of Public Health (INSP) institutional review boards. Enumerators visit participating households every 2 wk to collect diaries of purchases and receipts and register purchases by checking the pantry and a designated bin where the household members keep empty product packages. All items available with a barcode are scanned by the enumerator. The data for each purchase includes number of units, volume, price paid, and date of purchase.

Nielsen CPS samples households from 53 cities with >50,000 inhabitants and estimates weights for each household so that the sample is representative of the urban Mexican population.

From all households that participated in the Nielsen CPS in at least 2 mo during January 2012–December 2014, we excluded three households because of incomplete data on covariates. Our analytical sample includes 204,584 household-months, across 6,248 unique households. Average household follow-up was 32.7 mo; 78% participated in all 36 mo.

### Covariates

SES categories were based on those provided by The Nielsen Company, which are defined with a score system that classifies households in seven categories as proposed by the Mexican Association of Market Intelligence and Opinion. This measure of SES was validated and is the standard one used in market research in Mexico. The score considers the education level of the member with the largest household income contribution and seven household assets: number of rooms, type of floor, number of bathrooms, shower, gas range, number of light bulbs, and number of cars. The cutoff points for the seven categories are defined a priori to capture specific household characteristics and are not based on a population distribution; therefore, the sample in each category is not equal (e.g., the extreme categories combined have <10% of the sample). We classified SES as low (lower two categories), medium (middle three categories), and high (higher two categories). Additional variables include household composition (nine variables, each with the number of household members that were within each gender/age group [as presented in S1 Table]) and contextual measures (state-quarter unemployment rates [12] and minimum salary [13] adjusted by state-quarter consumer price index). See S1 Table for descriptive statistics on the sample.

### Food Categories

In this paper, we focus on volumes of overall taxed and untaxed foods and on subcategories of each. Classification of foods into untaxed and taxed categories was conducted by a team of registered dietitians from Mexico. In the case of law ambiguities for food classification, we consulted with the Ministry of Finances for clarification. For further description of each subcategory and the food classification process, see S2 Table.

Our analysis does not cover all food categories that households purchase; we did not include categories for which Nielsen CPS does not collect data or did not collect data consistently throughout the 36 months of the analysis. Examples of food categories not analyzed are chocolates, candies, and sweet bread from bakeries (taxed if energy density ≥275 kcal/100 g, though small bakeries were exempt from the tax in 2014), and unpackaged produce, tortillas, and unsweetened bread from bakeries (mainly untaxed).

### Statistical Analysis

All analyses were conducted in Stata, version 13 (College Station, TX). We first describe unadjusted, mean per capita volume purchases of taxed and untaxed foods (g/capita/month) from January 2012 to December 2014. Because the tax was implemented at one point in time across the entire country and, hence, we did not have a control population, we compared the purchases before and after the tax. Our pre-specified analytical strategy was based on the approach used by Colchero et al. in evaluating Mexico’s SSB tax [14] and in other research using longitudinal food purchase data to evaluate the effects of retailer- and industry-led initiatives, such as the United States Healthy Weight Commitment effort to reduce calories in the food supply [15,16]. Specifically, to account for the ongoing 2012–2013 trend, and to avoid assuming a decrease in purchases in 2014 was attributable to the tax if there was already a downward trend, we extrapolated with model predictions the 2012–2013 trend through 2014 and used it as our counterfactual (i.e., what was expected to happen without a tax in 2014 based on the 2012–2013 trend). We used a fixed effects model to predict the mean adjusted volume purchased in each month pre-tax, post-tax observed, and post-tax counterfactual. More detail about this pre-specified analytical strategy and deviations from this strategy are summarized in S1 Protocol.

The model specification was as follows:

$$Food_{hmy}=\alpha +\beta_{T}T_{my}+\beta_{S}S_{my}+\beta_{TS}(T_{my}*S_{my})+\beta_{y}Y_{y}+ϒ•H_{hy}+\phi •C_{my}+\lambda_{h}+\mu_{hmy}$$ Equation 1

The unit of analysis (g/capita/month) was the per capita volume of *Food* purchases in household *h*, month *m*, and year *y*. *T* is the post-tax period (0 = 2012–2013; 1 = 2014), *S* is 2nd semester (0 = Jan–Jun; 1 = Jul–Dec), *Y* is year (a continuous measure, 0 = 2012; 1 = 2013; 2 = 2014), *H* denotes the vector of year-specific household characteristics (SES and household composition), *C* denotes the vector of contextual measures (unemployment rates and minimum salary), and λ and *μ* are the error terms. The year slope reflects the change between 2012 and 2013 (not the change from 2012 through 2014, because the model is adjusting by post-tax period). Likewise, the post-tax coefficient estimates the change in 2014 beyond what was predicted in 2014 given the 2012–2013 slope. To assess changes within the year, we included a semester effect. We tried 2-, 3-, and 4-month periods, but due to high month-to-month variation and an unclear cyclic annual pattern in 2012’s and 2013’s purchases using shorter periods, we used a semester period (the second semester was always higher than the first in 2012 and 2013 for both taxed and untaxed foods). Regardless of the period length used in the model, the annual change remained unchanged. The *TS* interaction term allowed the semester effect to vary before and after the tax. Additionally, we controlled for the aforementioned household and contextual covariates.

Using this model, we predicted the mean adjusted volume purchased in each month pre-tax (2012–2013), post-tax observed (2014), and post-tax counterfactual (2014 but as if *T* = 0) to determine the absolute and relative differences over time. We present the predictions in the results section and regression coefficients in S3 Table.

Because the food purchase data had a skewed distribution, we tested a generalized linear model with log-link, which gives unbiased estimates [17]. Results from either a generalized linear model with log-link or a linear regression model were similar; hence, we used a linear regression to be able to use a fixed effects estimator. Fixed effects are advantageous because they control for unobservable time-invariant characteristics. We conducted this analysis separately for the taxed and untaxed categories in the entire sample.

We then performed analyses stratified by SES (low, medium, high) using the same specification as Eq 1 but without SES as a predictor variable. Stratified models allowed us to compare not only the tax effect, but also the absolute amount of purchases in each SES category. The SES coefficients in Eq 1 estimate the difference in the amount of purchases if a household changes SES category (intra-household), whereas our interest was on the difference in the amount of purchases across households with different levels of SES (inter-household).

Nearly all households purchased some food from untaxed (99.7% of households) and taxed (96%) foods each month. However, for subcategories, there was a large proportion of non-consumers. As a result, we used a two-part model [18] using probit and linear regression models with the same specification as above, except that the fixed effects estimator was not used.

The two-part model was as follows: Total amount of food subcategory purchase (g/capita/month) = [Probability of food subcategory purchase (probability/month)] * [Amount of food subcategory if purchased (g/capita/month)]

$$\begin{array}{l} FoodSub_{hmy}=Pr\left(FoodSub_{hmy}>0\right)*[FoodSub_{hmy}|Pr\left(FoodSub_{hmy}>0\right)]\\ Pr\left(FoodSub_{hmy}>0\right)=\alpha +\beta_{T}T_{my}+\beta_{s}S_{my}+\beta_{TS}\left(T_{my}*S_{my}\right)+\beta_{y}Y_{y}+ϒ•H_{hy}+\phi •C_{my}+\mu_{hmy}\\ FoodSub_{hmy}|Pr\left(FoodSub_{hmy}>0\right)=\alpha +\beta_{T}T_{my}+\beta_{s}S_{my}+\beta_{TS}\left(T_{my}*S_{my}\right)+\beta_{y}Y_{y}+ϒ•H_{hy}+\phi •C_{my}+\mu_{hmy} \end{array}$$ Equation 2

In all analyses, we used the household weights provided by Nielsen and estimated standard errors via bootstrapping by drawing 1,000 random samples with replacement with selection at the household level.

## Results

**Fig 1:**
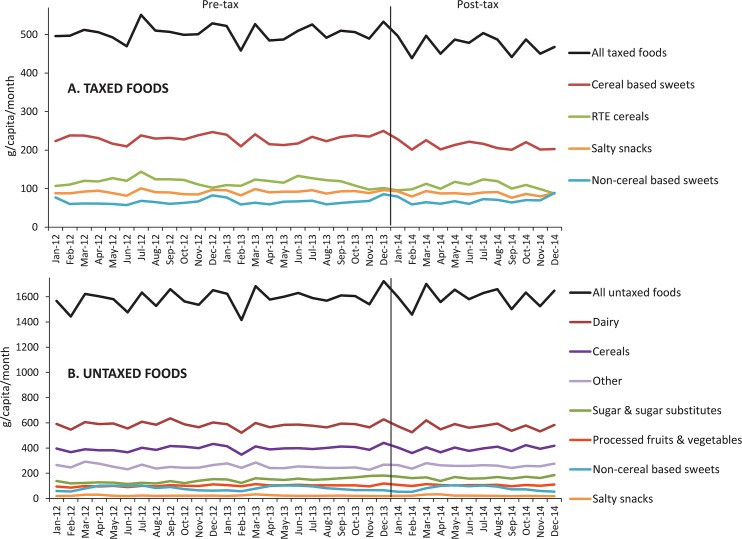
Monthly trends in unadjusted volume purchased (g/capita/month) of (A) taxed and (B) untaxed foods. Source: Authors’ own analyses and calculations based on data from Nielsen through its Mexico Consumer Panel Service (CPS) for the food and beverage categories for January 2012–December 2014.

Fig 1 shows the unadjusted mean volume trends for total taxed and untaxed food purchases and by subcategory. The 2012–2013 average of total volume of taxed food purchases was 505 g/capita/month, whereas the 2014 average was 474 g/capita/month, while the averages of total untaxed foods were 1,585 g/capita/month in 2012–2013 and 1,596 g/capita/month in 2014. As can be seen, purchases have high month-to-month variation.

Table 1 shows the adjusted absolute and relative differences between the counterfactual and observed volumes purchased in the post-tax period. On average, the total volume of taxed purchases had an absolute decline of 25 g per capita per month (*p* < 0.05), or a -5.1% relative change beyond what would have been expected based on pre-tax trends. The decline in volume of taxed purchases was 3.4% in the first semester of 2014 but growing to 6.7% in the second semester.

**Table 1:** Monthly average of predicted volume purchased (g/per capita) for taxed and untaxed food purchases.

	Taxed				Untaxed			
	Post-Tax Counterfactual (g/capita/month)	Post-Tax Observed (g/capita/month)	Observed vs. Counterfactual		Post-Tax Counterfactual (g/capita/month)	Post-Tax Observed (g/capita/month)	Observed vs. Counterfactual	
			Absolute Difference (g/capita/month)	% Difference			Absolute Difference (g/capita/month)	% Difference
	*Mean (95% Confidence Interval)*							
All								
Jan–Jun 2014	484 (467, 501)	467 (453, 482)	**-16 (-30, -3)**	-3.4%	1553 (1508, 1598)	1559 (1520, 1599)	6 (-27, 40)	0.4%
Jul–Dec 2014	500 (482, 517)	466 (452, 480)	**-34 (-48, -19)**	-6.7%	1585 (1539, 1631)	1568 (1527, 1609)	-17 (-53, 20)	-1%
All 2014	492 (475, 509)	467 (453, 480)	**-25 (-38, -12)**	-5.1%	1569 (1524, 1614)	1564 (1525, 1602)	-5 (-38, 27)	-0.3%
Low SES								
Jan–Jun 2014	430 (394, 466)	392 (367, 416)	**-38 (-66, -10)**	-8.9%	1248 (1163, 1332)	1245 (1177, 1312)	-3 (-63, 57)	-0.2%
Jul–Dec 2014	437 (401, 474)	387 (361, 413)	**-50 (-80, -20)**	-11.5%	1289 (1202, 1375)	1251 (1178, 1323)	-38 (-103, 28)	-2.9%
All 2014	434 (398, 469)	389 (365, 413)	**-44 (-72, -16)**	-10.2%	1268 (1184, 1353)	1248 (1180, 1315)	-20 (-79, 38)	-1.6%
Medium SES								
Jan–Jun 2014	480 (457, 504)	461 (442, 480)	**-19 (-37, -2)**	-4.1%	1557 (1493, 1620)	1533 (1483, 1583)	-24 (-73, 26)	-1.5%
Jul–Dec 2014	503 (477, 528)	465 (447, 483)	**-37 (-58, -17)**	-7.5%	1591 (1527, 1654)	1542 (1488, 1596)	-49 (-101, 4)	-3.1%
All 2014	491 (467, 515)	463 (446, 480)	**-28 (-46, -11)**	-5.8%	1574 (1511, 1636)	1537 (1488, 1587)	-36 (-83, 11)	-2.3%
High SES								
Jan–Jun 2014	564 (527, 602)	560 (530, 590)	-4 (-31, 23)	-0.8%	1902 (1813, 1991)	1946 (1865, 2027)	44 (-32, 120)	2.3%
Jul–Dec 2014	573 (533, 613)	551 (521, 580)	-22 (-51, 6)	-3.9%	1919 (1827, 2011)	1957 (1876, 2038)	38 (-43, 120)	2%
All 2014	569 (531, 607)	555 (527, 584)	-13 (-39, 12)	-2.3%	1911 (1821, 2000)	1952 (1874, 2029)	41 (-32, 115)	2.2%

Source: Authors’ own analyses and calculations based on data from Nielsen through its Mexico Consumer Panel Service (CPS) for the food and beverage categories for January 2012–December 2014.

Bold numbers: *p* < 0.05

**Fig 2:**
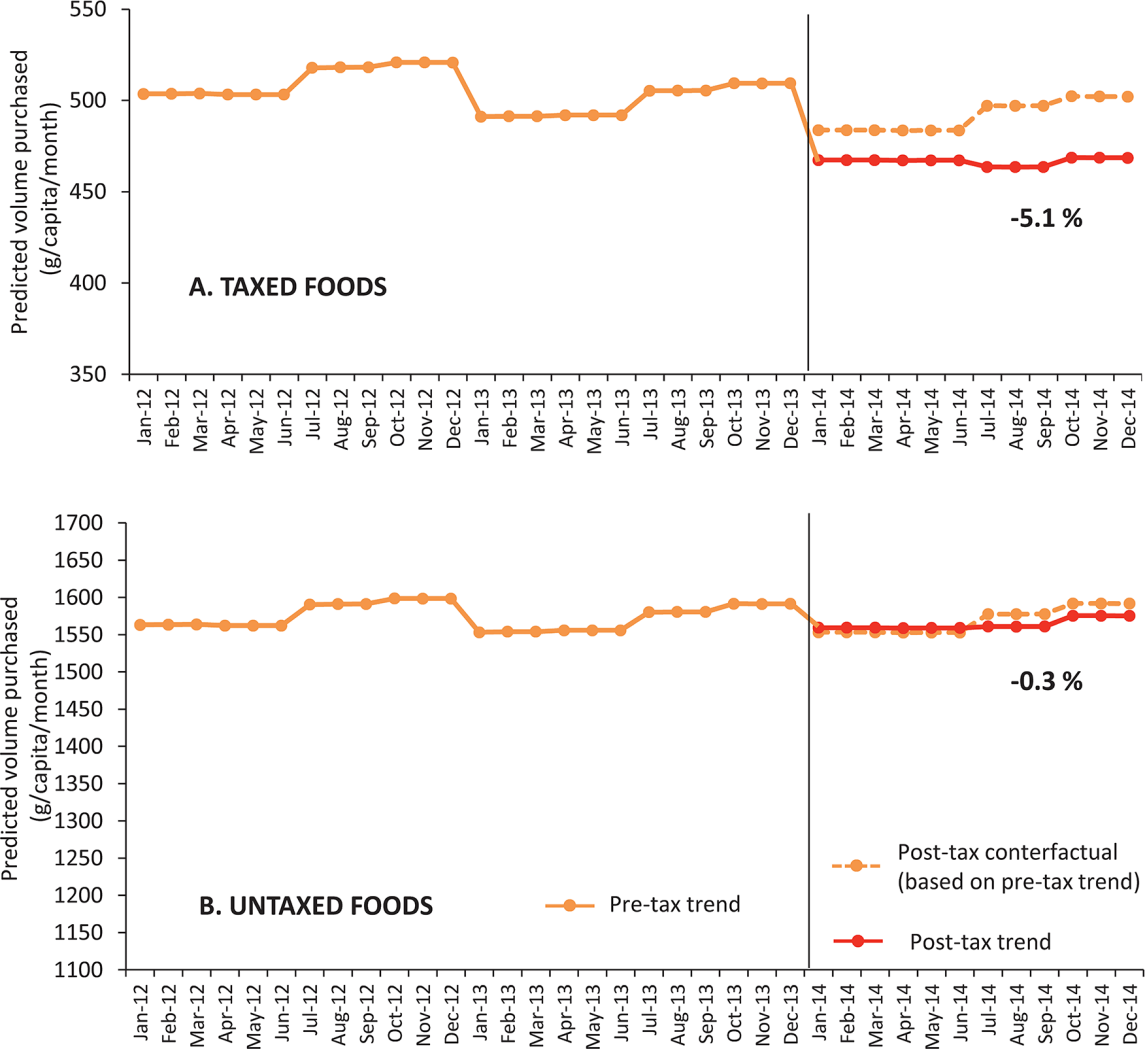
Monthly trends in predicted volume purchased (g/per capita) of (A) taxed and (B) untaxed foods compared to post-tax counterfactual. Source: Authors’ own analyses and calculations based on data from Nielsen through its Mexico Consumer Panel Service (CPS) for the food and beverage categories for January 2012–December 2014.

As can be seen in Fig 2, the effect during the second semester was larger because, based on previous trends, the purchases were expected to increase in the second semester, but they remained stable throughout 2014. No differences were detected for untaxed purchases.

Overall, low SES households bought less taxed food before and after the tax compared to their higher SES counterparts but showed the greatest response to the tax (Table 1, S1 Fig). On average, in 2014, low SES households purchased 10.2% less taxed foods than expected (*p* < 0.05), whereas medium SES households purchased 5.8% less taxed foods (*p* < 0.05), and high-income households’ purchases did not change. Table 2 (S2 and S3 Figs) shows the subcategories of taxed and untaxed food purchases from the two-part model. The total amount purchased is estimated by multiplying the probability of any purchase during a month by the amount purchased in months with purchases higher than zero. In Table 2, we present the predicted probability, amount, and total, whereas in S2 and S3 Figs, we only present the total. The greatest changes in total purchases were observed among taxed salty snacks (-6.3% beyond expected, *p* < 0.05) and taxed cereal-based sweets (-5.2% beyond expected, *p* < 0.05), while taxed non-cereal-based sweets and ready-to-eat cereals did not change. Interestingly, in the case of taxed salty snacks, what drove the overall change was a change in the probability of purchasing.

**Table 2:** Monthly average of predicted volume purchased (g/per capita) for the post-tax taxed and untaxed food subcategories.

	Probability				Amount				Total			
	Post-Tax Counterfactual (Probability/ Month)	Post-Tax Observed (Probability/Month)	Observed vs. Conterfactual		Post-Tax Counterfactual (g/capita/month)	Post-Tax Observed (g/capita/ month)	Observed vs. Conterfactual		Post-Tax Counterfactual (g/capita/month)	Post-Tax Observed (g/capita/ month)	Observed vs. Conterfactual	
			Absolute Difference (Probability/ Month)	Percent Difference			Absolute Difference (g/capita/ month)	Percent Difference			Absolute Difference (g/capita/ month)	Percent Difference
	*Mean (95% Confidence Interval)*											
Taxed												
Salty snacks	0.78 (0.76, 0.79)	0.72 (0.71, 0.74)	**-0.05 (-0.06, -0.04)**	-6.5%	123 (116, 130)	124 (118, 129)	0 (-5, 6)	0.4%	94 (88, 100)	88 (84, 93)	**-6 (-11, -1)**	-6.3%
Cereal-based sweets	0.87 (0.86, 0.88)	0.84 (0.84, 0.85)	**-0.02 (-0.03, -0.01)**	-2.7%	267 (254, 279)	260 (251, 269)	-7 (-16, 3)	-2.5%	230 (219, 242)	218 (210, 227)	**-12 (-21, -3)**	-5.2%
Ready-to-eat cereals	0.56 (0.54, 0.58)	0.54 (0.53, 0.55)	**-0.02 (-0.04, -0.01)**	-4%	210 (200, 219)	215 (208, 222)	5 (-4, 14)	2.5%	119 (112, 126)	117 (112, 122)	-2 (-8, 4)	-1.6%
Non-cereal-based sweets	0.52 (0.5, 0.54)	0.49 (0.48, 0.51)	**-0.03 (-0.04, -0.01)**	-5.1%	138 (131, 145)	143 (138, 149)	6 (-1, 12)	4.2%	71 (66, 75)	70 (67, 73)	-1 (-5, 3)	-1.2%
Untaxed												
Sugar and sugar substitutes	0.37 (0.35, 0.39)	0.35 (0.34, 0.36)	**-0.02 (-0.04, 0.00)**	-5.9%	496 (464, 528)	481 (455, 507)	-15 (-50, 20)	-3.1%	182 (167, 198)	166 (154, 178)	**-16 (-31, -1)**	-8.9%
Cereals	0.94 (0.93, 0.94)	0.94 (0.93, 0.94)	0.00 (-0.01, 0.01)	0.0%	439 (423, 455)	436 (424, 447)	-4 (-17, 10)	-0.8%	410 (394, 426)	406 (395, 418)	-3 (-16, 10)	-0.8%
Dairy	0.96 (0.95, 0.96)	0.95 (0.95, 0.96)	0.00 (-0.01, 0.00)	-0.5%	595 (572, 618)	603 (586, 620)	8 (-10, 27)	1.4%	569 (545, 592)	574 (557, 591)	5 (-13, 24)	0.9%
Processed fruits and vegetables	0.46 (0.44, 0.48)	0.47 (0.45, 0.48)	0.01 (-0.01, 0.02)	1.8%	245 (227, 263)	247 (236, 259)	2 (-11, 15)	0.9%	116 (105, 127)	119 (111, 127)	3 (-5, 11)	2.6%
Salty snacks	0.33 (0.32, 0.35)	0.32 (0.31, 0.33)	**-0.02 (-0.03, 0.00)**	-5.0%	68 (63, 73)	73 (70, 76)	5 (1, 10)	7.7%	22 (20, 24)	23 (21, 24)	1 (-1, 2)	2.7%
Non-cereal-based sweets	0.39 (0.37, 0.41)	0.39 (0.38, 0.4)	0.00 (-0.01, 0.02)	0.5%	203 (187, 219)	217 (206, 227)	14 (-2, 29)	6.7%	80 (73, 88)	86 (81, 91)	6 (-2, 13)	7.2%
Other	0.85 (0.84, 0.86)	0.86 (0.85, 0.86)	0.01 (-0.01, 0.02)	0.6%	290 (279, 301)	311 (302, 320)	21 (11, 30)	7.2%	245 (234, 255)	264 (256, 272)	**19 (11, 28)**	8.0%

Bold numbers *p* < 0.05

Source: Authors’ own analyses and calculations based on data from Nielsen through its Mexico Consumer Panel Service (CPS) for the food and beverage categories for January 2012–December 2014.

Among untaxed foods, there were only significant declines in the volume of sugar and sugar substitutes purchased compared to what was expected based on the pre-tax trend (-8.9%, *p* < 0.05), though the absolute volume of purchases continued to increase. In other words, the upward trend in the volume of sugar and sugar substitutes observed in 2012–2013 continued in 2014, but was smaller than expected (see S3 Fig). Purchases of the food group “other” increased by 8.0% (*p* < 0.05).

## Discussion

For the first full year after Mexico’s taxes on SSBs and nonessential energy-dense food taxes, we find significant changes in the observed per capita volume of household purchases of taxed foods compared to the counterfactual (i.e., what was expected based on pre-tax trends). Overall, we find that taxed foods declined by 25 g/capita/month (-5.1%), whereas untaxed food purchases did not change (-0.3%). Moreover, we find much larger declines for lower SES households (-10.2%), whereas medium SES households changed by 5.8% and high SES households did not change.

Empirical evidence on the effect of food and nutrient taxes is limited. With regards to Denmark’s short-lived saturated fat tax, one study of household food purchases found a 10%–15% reduction in purchases of butter, blends, margarines, and oils in the first 9 mo of implementation, when the increase in price of these products was 8%–22% [10]. A recent evaluation using cruder expenditure data from an income and expenditure national survey of the Hungarian tax on foods high in salt, sugar, or caffeine found a 3.4% decrease in the volume purchased of processed foods after the tax, with no corresponding change in unprocessed foods [11], though these processed food categories were not necessarily reflective of taxed versus untaxed foods. The present results show that, at least in Mexico, a relatively modest tax can, in the short run, result in a substantial decline in volume purchased of taxed foods. However, it is important to consider that taxes could affect purchases with other mechanisms in addition to the increase of price. Press coverage or public discussion of the tax can help discourage the consumption of the taxed products in the population; but, for the nonessential energy-dense tax in Mexico, the coverage has been small relative to the SSB tax [19,20]. On the other hand, the presence of the SSB tax could have had an effect on the purchases of nonessential energy dense foods, because these items might be complementary and consumed together. Previous estimations of SSB own and cross-price elasticities in Mexico reported that for a 1% increase in the price of SSB, the purchase of candies and snacks would decrease 0.44% and 0.23%, respectively, and that for a 1% increase in the price of candies and snacks, these would decrease 1.15% and 0.98%, respectively [21]. Therefore, it is likely that the decrease we found in taxed foods is due to both the SSB and the nonessential energy-dense food taxes.

The reduction of 25 g/capita/month represents 70 to 110 kcal (energy density is at least 275 kcal/100 g, but based on the ENSANUT 2012, the mean energy density for the intake of taxed foods is 430 kcal/100 g). Although in absolute terms this reduction is small, the purchases captured in Nielsen only represent a fraction of all household purchases, and real absolute change in energy intake from taxed food might be larger.

The changes in taxed foods were for salty snacks and cereal-based sweets. Interestingly, for salty snacks, all the change was due to changes in probability of purchasing, suggesting that, for this item, people prefer to decrease the frequency of purchases rather than the amount. Moreover, we saw smaller-than-expected increases in the volume of sugar and sugar substitutes, suggesting that households are not necessarily substituting sugary home-prepared foods or beverages for pre-packaged taxed sweets.

Lower SES households were more responsive to the tax than middle SES households, while higher SES households showed no statistically significant change in purchases, consistent with results of the evaluation of Mexico’s SSB tax [22]. This is important, considering that in Mexico, although lower SES groups still have slightly lower prevalence of obesity and diabetes [23], the costs associated with obesity and its comorbidities represent a higher proportion of their income. In other countries such as the US, where obesity prevalence is highest among people with low SES, a similar response to such a tax could lead to decreased disparities in diet and obesity. Long-term effects must be monitored, as we expect the industry to develop strategies in response to the tax, including product reformulation. For example, in the jam and spreads categories, we found that in 2014, a number of products were reformulated to fall under the 275 kcal/100 g threshold. The authors of the evaluation of Hungary’s junk food tax also reported that a sizable proportion (40%) of Hungary’s food manufacturers reported reformulating products to avoid taxation [11].

A great complexity of implementing a food tax is to define the characteristics of the foods subject to it. If only selected unhealthy foods are taxed, individuals can substitute with other unhealthy untaxed foods; on the other hand, if the tax categorization is too broad, many relatively healthy products will also be affected, increasing the cost of food without the public health benefit [24,25]. Overall, this tax successfully targeted unhealthy foods, as it focused on processed foods and did not disincentive traditional cooking ingredients such as sugar and fats (a criticism the Danish fat tax has received) [26]. However, the use of a single energy-dense cut-point in the Mexican tax without other nutritional attributes left out foods that are otherwise considered unhealthy (e.g., most ice creams were untaxed), whereas foods like peanuts and nuts were taxed. Moreover, sorting products out into “essential” versus “nonessential” is an iterative process, and throughout 2014 there were clarifications on the initial law ambiguities, representing about 2.3% of all products (see S2 Table). In contrast, new Chilean controls on food marketing that will go into effect July 1, 2016, uses as a cutoff not only energy but also sodium, saturated fat, and total sugar for foods and beverages separately [27]. An additional complexity of analyzing the Mexican tax is that each producer interprets the law and determines the total amount they have to pay (without reporting for which products they are paying). Thus, we cannot be certain which exact products were actually taxed.

This work had several important limitations. First, we were unable to capture and analyze all foods that households purchased, including unpackaged produce, chocolates, candies, tortilla, and bread from bakeries. However, even for foods that were collected consistently in the Nielsen CPS, we captured only 474 g/capita/month of taxed foods in 2014. This is lower than what we would expect an average person to purchase, particularly if we compare to Euromonitor retail sales of 1,236 g/capita/month (excluding chocolates and bread from bakeries) or to the National Institute of Statistics and Geography’s (INEGI’s) manufacturer’s industry survey of 1245 g/capita/month (excluding chocolate) (S4 and S5 Tables). Similar to other consumer panel surveys, it is expected that purchases from Nielsen CPS would be lower, because INEGI’s data is of total sales (including food services and exports), and also because Euromonitor and INEGI use aggregate food categories that include untaxed items. Still, we are missing some amount of food purchases, most likely items purchased and consumed away from home. It is possible that the items not captured in the Nielsen dataset have a different trend than that found in our results. However, as can be seen in S4 and S5 Tables, INEGI’s and Euromonitor’s sales also display a decrease of 4.2% to 6.2% compared to 2013 for taxed foods and no change or slight increase for untaxed foods.

Our model and counterfactual comparisons allowed us to examine what happened post-tax compared to what would have happened if the pre-tax trends had continued. However, this comparison assumes that pre-tax trends would have continued, which may not have been the case, and we cannot rule out that these results may have been influenced by other concomitant changes unrelated to taxes, including economic trends and anti-obesity and public health campaigns and regulations. [28]. Another limitation is that we only have 2 y of data prior to the tax. The discussion of the SSB tax and the overall obesity issue has intensified since late 2012 [19]. Capturing the effect of tax discussions on purchases beyond the effect of the tax itself is of interest; however, we do not have data before 2012 to use as a comparison and to be able to assess this. Finally, our sample was only representative of urban Mexican households in cities with more than 50,000 inhabitants. This sample represents 63% of the Mexican population and 75% of food and beverage expenditures [29], but we do not know if rural households responded differently to these taxes. Regardless, this study provides the first snapshot of overall trends in food purchasing a year after the nonessential food tax was passed. Future work will extend this analysis by examining changes in the nutrient profile of nonessential foods in response to this tax, including sugar, saturated fat, energy density, and sodium.

## Conclusion

This evaluation of Mexico’s nonessential food and SSB taxes shows that the volume of taxed food purchases declined over what was expected, and that these results were similar in direction and magnitude to declines in SSBs in response to the SSB tax. Declines after the tax were statically significant among low and medium SES households and for selected food subcategories (salty snacks and cereal-based sweets). Our results can orient Mexican policymakers, who every year decide on the continuation of the tax, as well as policymakers from others countries currently considering the implementation of foods taxes. However, the impact of this tax on overall energy intake, dietary quality, and food purchase patterns, as well as how these changes relate to weight status, remains to be studied.

## Supporting Information

S1 Abstract Spanish abstract. (DOCX)

S1 Fig Monthly trends in predicted volume purchased (g/capita/month) of (A) taxed and (B) untaxed foods comparing to post-tax counterfactual by SES.
(DOCX)

S2 Fig Monthly trends in predicted total volume purchased (g/capita/month) of taxed food subcategories: (A) salty snacks, (B) cereal-based sweets, (C) ready-to-eat cereals, (D) non-cereal-based sweets.
(DOCX)

S3 Fig Monthly trends in predicted total volume purchased (g/capita/month) of untaxed food subcategories: (A) sugar and sugar substitutes, (B) cereals, (C) dairy, (D) processed fruits and vegetables, (E) salty snacks, (F) non-cereal-based sweets, (G) other.
(DOCX)

S1 Protocol Summary of the pre-specified analysis plan and deviations. (DOCX)

S1 Table Weighted sample characteristics of Mexico Nielsen Consumer Panel Service by year. (DOCX)

S2 Table Examples of food items for each food subcategory and details on food classification process. (DOCX)

S3 Table Main regression coefficients of the models used to predict volume purchased. (DOCX)

S4 Table National sales of mainly taxed foods in 2014 and percent change in sales from previous years reported by INEGI and Euromonitor. (DOCX)

S5 Table National sales of mainly untaxed foods in 2014 and percent change in sales from previous years reported by INEGI and Euromonitor. (DOCX)

## Abbreviations

CPS: Consumer Panel Services
ENSANUT: National Health and Nutrition Survey
INEGI: National Institute of Statistics and Geography
INSP: National Institute of Public Health
IRB: institutional review board
SES: socioeconomic status
SSB: sugar-sweetened beverage

## References

[1] Rivera JA, de Cossio TG, Pedraza LS, Aburto TC, Sanchez TG, Martorell R. Childhood and adolescent overweight and obesity in Latin America: a systematic review. Lancet Diabetes Endocrinol. 2014;2(4):321–32. 10.1016/S2213-8587(13)70173-6 24703050

[2] Barquera S, Campos-Nonato I, Hernández-Barrera L, Pedroza A, Rivera-Dommarco JA. Prevalence of obesity in Mexican adults 2000–2012. Salud publica de Mexico. 2013;55:S151–S60. 24626691

[3] Villalpando S, de la Cruz V, Rojas R, Shamah-Levy T, Ávila MA, Gaona B, et al Prevalence and distribution of type 2 diabetes mellitus in Mexican adult population: a probabilistic survey. Salud Pública de Méx. 2010;52:S19–S26. 10.1590/s0036-36342010000700005 20585724

[4] Stern D, Piernas C, Barquera S, Rivera JA, Popkin BM. Caloric Beverages Were Major Sources of Energy among Children and Adults in Mexico, 1999–2012. J Nutr. 2014;144(6):949–956. 10.3945/jn.114.190652 24744311 PMC4083240

[5] Barquera S, Campirano F, Bonvecchio A, Hernández L, Rivera J, Popkin B. Caloric beverage consumption patterns in Mexican children. Nutr J. 2010;9:47–56. 10.1186/1475-2891-9-47 20964842 PMC2987771

[6] Barquera S, Hernandez-Barrera L, Tolentino ML, Espinosa J, Ng SW, Rivera JA, et al Energy intake from beverages is increasing among Mexican adolescents and adults. J Nutr. 2008;138(12):2454–61. 10.3945/jn.108.092163 19022972

[7] Hawkes C. Uneven dietary development: linking the policies and processes of globalization with the nutrition transition, obesity and diet-related chronic diseases. Global Health. 2006;2(1):4. 16569239 10.1186/1744-8603-2-4 PMC1440852

[8] Pan American Health Organization. Ultra-processed food and drink products in Latin America: Trends, impact on obesity, policy implications. 2015. http://www.paho.org/hq/index.php?option=com_content&view=article&id=11153%3Aultra-processed-food-and-drink-products&catid=4999%3Aactive-living-documents&lang=en

[9] Pedraza L, Aburto T, Sánchez T, Rivera J. Contribution of food groups to the total dietary energy intake of Mexican children, adolescents and adults. FASEB J. 2014;28:393.3

[10] Jensen JD, Smed S. The Danish tax on saturated fat–Short run effects on consumption, substitution patterns and consumer prices of fats. Food Policy. 2013;42(0):18–31.

[11] Bíró A. Did the junk food tax make the Hungarians eat healthier? Food Policy. 2015;54:107–15.

[12] Instituto Nacional de Estadística y Geografia, empleo Endoy. Indicadores Estratégicos 2014 [cited July 2015]. http://www3.inegi.org.mx/sistemas/temas/default.aspx?s=est&c=25433&t=1.

[13] Comisión Nacional de los Salarios Mínimos. Tabla de Salarios Mínimos Generales y Profesionales por áreas geográficas [cited July 2015]. http://www.conasami.gob.mx/t_sal_mini_prof.html.

[14] Colchero A, Rivera JA, Popkin BM, Ng SW. Beverage purchases from stores in Mexico under the excise tax on sugar sweetened beverages: observational study. BMJ. 2016;352(h6704). 10.1136/bmj.h6704 PMC4986313 26738745

[15] Ng SW, Popkin BM. The Healthy Weight Commitment Foundation Pledge. Am J Prev Med.47(4):520–30. 10.1016/j.amepre.2014.05.030 25240968 PMC4171651

[16] Taillie L, Ng S, Popkin B. Gains made by Walmart’s healthier food initiative mirror preexisting trends. Health Affair. 2015;34(11):1869–76. 10.1377/hlthaff.2015.0072 PMC4692370 26526244

[17] Manning WG, Mullahy J. Estimating log models: to transform or not to transform? Journal of health economics. 2001;20(4):461–94. 11469231 10.1016/s0167-6296(01)00086-8

[18] Belotti F, Deb P, Manning WG, Norton EC. twopm: Two-part models. Stata Journal. 2015;15(1):3–20.

[19] Donaldson E. Advocating for sugar-sweetened beverage taxation: a case study of Mexico 2015 [September 1, 2015]. http://www.jhsph.edu/departments/health-behavior-and-society/_pdf/Advocating_For_Sugar_Sweetened_Beverage_Taxation.pdf.

[20] Cooper B. How soda is drawing fire from food in Mexico obesity battle. Just Food. August 24, 2015. [cited 2016 March 25]. http://www.just-food.com/analysis/how-soda-is-drawing-fire-from-food-in-mexico-obesity-battle_id130918.aspx.

[21] Colchero M, Salgado J, Unar M, Hernández-Ávila M, Rivera-Dommarco J. Price elasticity of the demand for sugar sweetened beverages and soft drinks in Mexico. Economics & Human Biology. 2015;19:129–37. 26386463 10.1016/j.ehb.2015.08.007

[22] Colchero A, Rivera JA, Popkin BM, Ng SW. Beverage purchases from stores since the start of the Mexican Sugar-Sweetened Beverage excise tax: A year out. BMJ. 2015;In press. 10.1136/bmj.h6704 PMC4986313 26738745

[23] Rivera Dommarco JA, Campos-Nonato I, Barquera Cervera S, Gonzalez de Cossio T. Epidemiología de la obesidad en México: magnitud, distribución, tendencias y factores de riesgo Obesidad en México: recomendaciones para una política de Estado. Mexico: Universidad Nacional Autónoma de México; 2012.

[24] Cash S, Lacanilao RD. Taxing food to improve health: economic evidence and arguments. J Agr Resour Econ. 2007;36(2):174.

[25] Andreyeva T, Long MW, Brownell KD. The impact of food prices on consumption: A systematic review of research on the price elasticity of demand for food. Am J Public Health. 2009;100(2):216–22. 10.2105/AJPH.2008.151415 20019319 PMC2804646

[26] Brunner-Brown JA. “Fat Taxes” Fighting Globesity Ignore Food Demand Inelasticities. Ann Surv Int'l & Comp L. 2015;20(1):13.

[27] Popkin BM, Hawkes C. The sweetening of the global diet, particularly beverages: patterns, trends and implications for diabetes prevention. Lancet Diabetes Endocrinol. 2015;4(2):174–86. 10.1016/S2213-8587(15)00419-2 26654575 PMC4733620

[28] Secretaría de Salud. Estrategia Nacional para la Prevención y el Control del Sobrepeso, la Obesidad y la Diabetes. [National Strategy for the Prevention and Control of Overweight, Obesity and Diabetes] Mexico, D.F.; 2013.

[29] Instituto Nacional de Estadística y Geografia. Encuesta Nacional de Ingreso y Gastos de los Hogares. [National Household Income and Expenditure Survey]. 2008, 2010, 1012, 1014 [cited 2015 July 1]. www.inegi.org.mx/est/contenidos/Proyectos/encuestas/hogares/regulares/enigh.

