# Supplementary material for: First-Year Evaluation of Mexico’s Tax on Nonessential Energy-Dense Foods: An Observational Study
Source: PLoS Med. 2016 Jul 5;13(7):e1002057. doi: 10.1371/journal.pmed.1002057 (PMC4933356; doi:10.1371/journal.pmed.1002057)
Supplement: S1 Abstract — (DOCX) [file pmed.1002057.s001.docx]

**S1 Abstract in Spanish**

**Resumen**

**Introducción:** En un esfuerzo por prevenir el continuo incremento en obesidad y diabetes, en Enero de 2014, el gobierno Mexicano implementó un impuesto de 8% a los alimentos no básicos con densidad energética ≥275 kcal/100g y un impuesto de un peso por litro a las bebidas azucaradas. A la fecha existen pocas evaluaciones rigurosas de los impuestos a alimentos en el mundo. El objetivo de este estudio fue identificar los cambios en el volumen de compras de alimentos empaquetados con y sin impuesto, en la muestra completa y por nivel socioeconómico (NSE).

**Métodos y Resultados:** Este estudio se basa en datos de compras de alimentos empaquetados de hogares representativos de la población urbana de México provenientes del Panel de Consumidores de Nielsen México. Incluimos 6248 hogares que participaron en el panel al menos durante dos meses entre 2012 y 2013; el promedio de seguimiento fue 32.7 meses. Analizamos el volumen de compras de alimentos con y sin impuesto de Enero de 2012 a Diciembre de 2014 con un modelo longitudinal, de efectos fijos, que ajustó por la tendencia previa y así estimar si la tendencia observada después del impuesto era significativamente diferente que la tendencia esperada en base a la tendencia previa al impuesto. Ajustamos por las características del hogar y factores contextuales como salario mínimo y tasa de desempleo. El volumen promedio de compras de alimentos con impuesto en 2014 cambió -25 (Intervalo de Confianza 95% = -46, -11) g per cápita al mes, es decir, un cambio de 5.1% más allá de lo que se hubiera esperado con base en la tendencia previa al impuesto (2012-2013). No hubo cambio en la compra de los alimentos sin impuesto. Los hogares con NSE bajo compraron en promedio 10.2% menos alimentos con impuesto que lo esperado [-44 (-72, -16) g per cápita al mes]; los hogares de NSE medio compraron 5.8% menos alimentos con impuesto que lo esperado [-28 (-46, -11) g per cápita al mes]; mientras que los hogares con NSE alto no presentaron cambios. Las limitaciones principales de nuestros resultados son: la incapacidad de inferir causalidad porque los impuestos se implementaron a nivel nacional (no hay grupo control), la muestra es representativa únicamente de las áreas urbanas, el contar solamente con dos años de datos previo al impuesto, y que como ocurre con todos los paneles de consumidores no pudimos captar el total de las compras de alimentos del hogar.

**Conclusiones:** Las compras de los hogares de alimentos no básicos con alta densidad energética disminuyeron en el primer año después de la implementación de los impuestos a estos alimentos y a las bebidas azucaradas en México. Futuros estudios deben evaluar el impacto de los impuestos en el consumo energético total, la calidad de la dieta y en los patrones de compras totales.
