# Supplementary material for: First-Year Evaluation of Mexico’s Tax on Nonessential Energy-Dense Foods: An Observational Study
Source: PLoS Med. 2016 Jul 5;13(7):e1002057. doi: 10.1371/journal.pmed.1002057 (PMC4933356; doi:10.1371/journal.pmed.1002057)

**S1 Fig.** Monthly trends in predicted volume purchased (g/capita/month) of A) taxed and B) untaxed foods comparing to post-tax counterfactual by SES.

Source: Authors’ own analyses and calculations based on data from Nielsen through its Mexico Consumer Panel Service (CPS) for the food and beverage categories for January 2012 – December 2014.


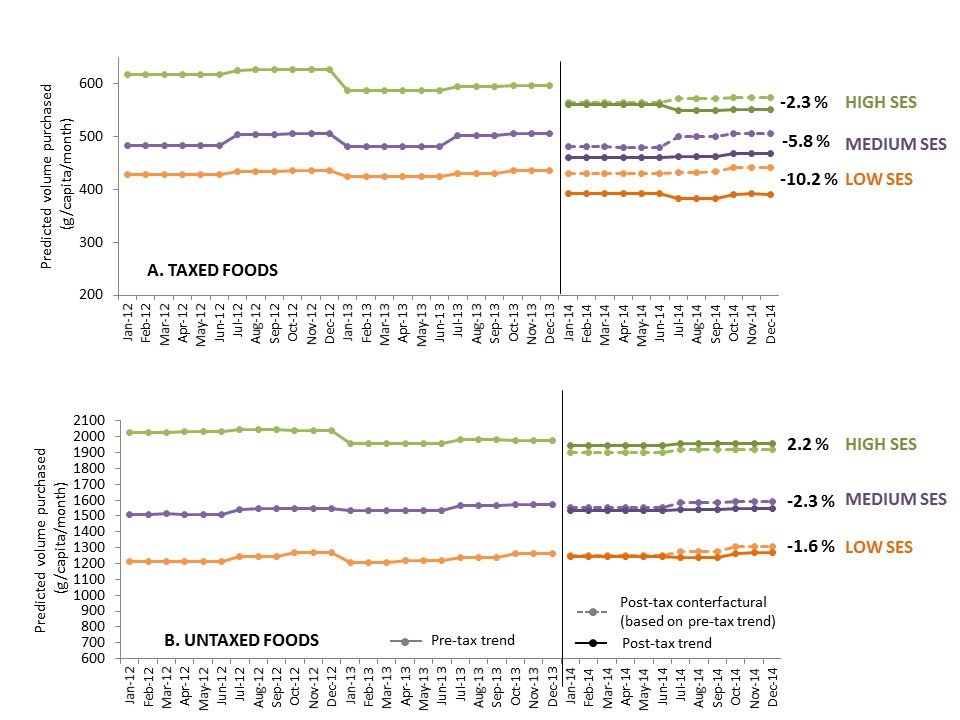

Supplement: S1 Fig — Monthly trends in predicted volume purchased (g/capita/month) of (A) taxed and (B) untaxed foods comparing to post-tax counterfactual by SES. (DOCX) [file pmed.1002057.s002.docx]
