# Supplementary material for: First-Year Evaluation of Mexico’s Tax on Nonessential Energy-Dense Foods: An Observational Study
Source: PLoS Med. 2016 Jul 5;13(7):e1002057. doi: 10.1371/journal.pmed.1002057 (PMC4933356; doi:10.1371/journal.pmed.1002057)

**S2 Fig.** Monthly trends in predicted total volume purchased (g/capita/month) of taxed food subcategories: A) salty snacks, B) cereal based sweets, C) ready-to-eat cereals, D) non-cereal based sweets.

Source: Authors’ own analyses and calculations based on data from Nielsen through its Mexico Consumer Panel Service (CPS) for the food and beverage categories for January 2012 – December 2014.


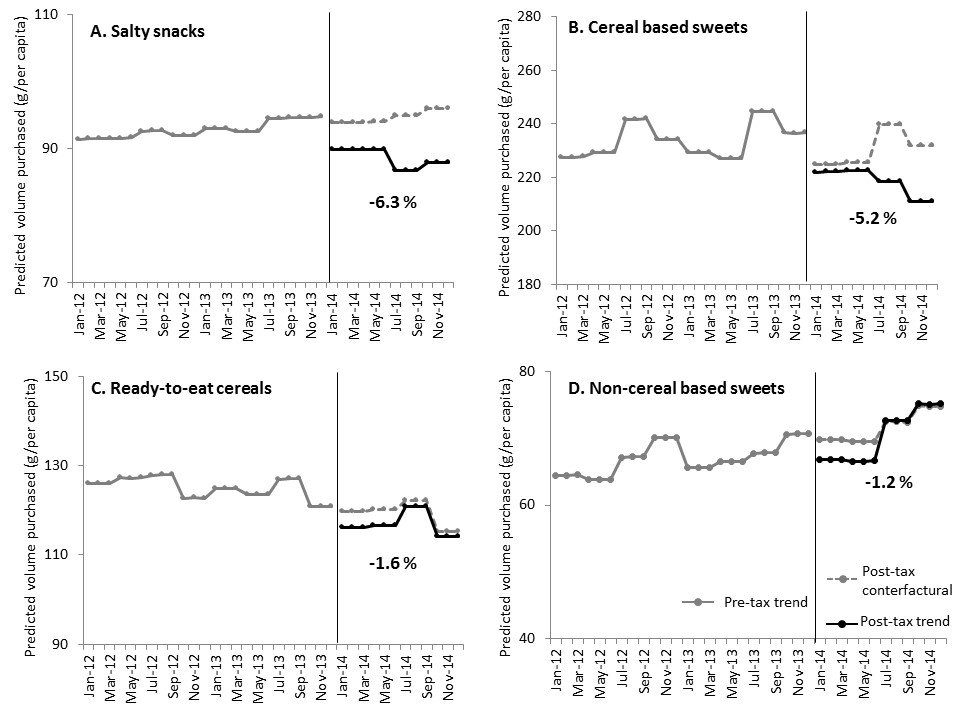

Supplement: S2 Fig — Monthly trends in predicted total volume purchased (g/capita/month) of taxed food subcategories: (A) salty snacks, (B) cereal-based sweets, (C) ready-to-eat cereals, (D) non-cereal-based sweets. (DOCX) [file pmed.1002057.s003.docx]
