# Supplementary material for: First-Year Evaluation of Mexico’s Tax on Nonessential Energy-Dense Foods: An Observational Study
Source: PLoS Med. 2016 Jul 5;13(7):e1002057. doi: 10.1371/journal.pmed.1002057 (PMC4933356; doi:10.1371/journal.pmed.1002057)

**S3 Fig.** Monthly trends in predicted total volume purchased (g/capita/month) of untaxed food subcategories: A) sugar and sugar substitutes, B) cereals, C) dairy, D) processed fruits and vegetables, E) salty snacks, F) non-cereal based sweets, G) other.

Source: Authors’ own analyses and calculations based on data from Nielsen through its Mexico Consumer Panel Service (CPS) for the food and beverage categories for January 2012 – December 2014.


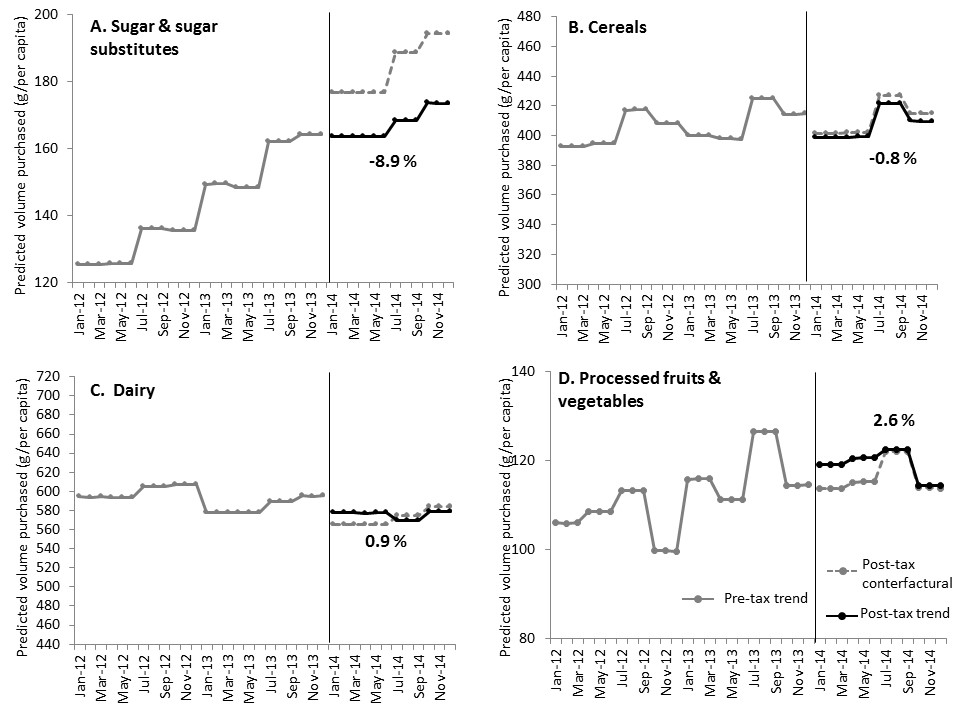


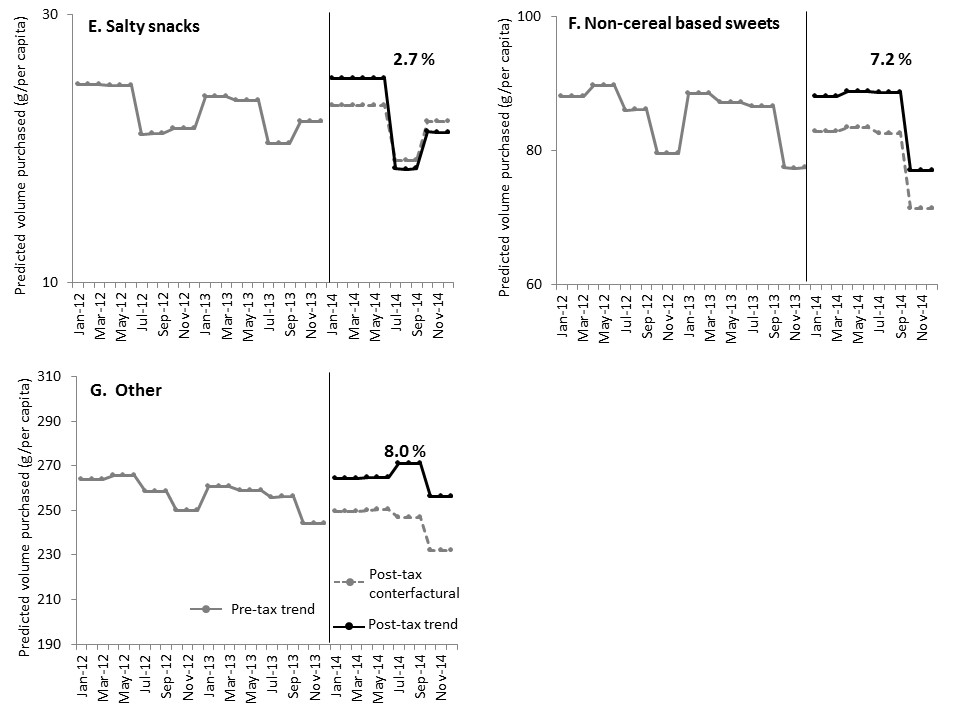

Supplement: S3 Fig — Monthly trends in predicted total volume purchased (g/capita/month) of untaxed food subcategories: (A) sugar and sugar substitutes, (B) cereals, (C) dairy, (D) processed fruits and vegetables, (E) salty snacks, (F) non-cereal-based sweets, (G) other. (DOCX) [file pmed.1002057.s004.docx]
