# Supplementary material for: First-Year Evaluation of Mexico’s Tax on Nonessential Energy-Dense Foods: An Observational Study
Source: PLoS Med. 2016 Jul 5;13(7):e1002057. doi: 10.1371/journal.pmed.1002057 (PMC4933356; doi:10.1371/journal.pmed.1002057)
Supplement: S1 Protocol — (DOCX) [file pmed.1002057.s005.docx]

**S1 Protocol**

**Summary of the pre-specified analysis plan and deviations**

As stated in the text, our analytical strategy was based on the strategy used by Colchero et al. in evaluating Mexico’s SSB tax (14, 15). This strategy extrapolates pre-tax trends (2012-2013) to the post-tax period (2014) to model the expected trends in food purchases had a tax not occurred (i.e., the counterfactual). We then compare observed post-tax purchases to counterfactual post-tax purchases, controlling for existing trends, household-level characteristics, and other factors such as unemployment rate, to test whether any decline in the purchase of taxed foods was greater than expected based on pre-tax trends. We used a fixed effects model to predict the mean adjusted volume purchased in each month pre-tax, post-tax observed and post-tax counterfactual. The general equation used is stated in the text.

There were no major departures from the pre-specified analysis plan. To check that this plan was appropriate for the research question and did not violate statistical assumptions, we tested different model specifications to determine best model fit (e.g. GLM with log-link vs. linear model; 6-month, 3-month, vs. 4-month indicators; linear term at the month level vs. linear term at the year level). The overall annual effect of the tax remained unchanged regardless of the model used. For subcategories, given the large proportion of non-consumers we planned to use a two-part model with no fixed effects. Upon examining changes in probability of purchase of food subcategories as well as the amount of food purchased, we confirmed that the two-part model provided the best method to account for these correlated components of probability and amount purchased.
