# Supplementary material for: First-Year Evaluation of Mexico’s Tax on Nonessential Energy-Dense Foods: An Observational Study
Source: PLoS Med. 2016 Jul 5;13(7):e1002057. doi: 10.1371/journal.pmed.1002057 (PMC4933356; doi:10.1371/journal.pmed.1002057)
Supplement: S1 Table — (DOCX) [file pmed.1002057.s006.docx]

| **S1 Table.** Weighted sample characteristics^1^ of Mexico Nielsen Consumer Panel Service by year. | | | |
| --- | --- | --- | --- |
|  | **2012** | **2013** | **2014** |
| Sample households, n | 5,785 | 5,750 | 5,652 |
| Sample household-months, n | 68,621 | 68,579 | 67,384 |
| Household socio-economic status, % |  |  |  |
| Low | 20 | 23.4 | 25 |
| Middle | 58.1 | 52.6 | 51.3 |
| High | 21.9 | 24.1 | 23.6 |
| Household composition, number of members (mean ± SD) |  |  |  |
| Children 0-1 y | 0.37 ± 0.63 | 0.24 ± 0.51 | 0.10 ± 0.34 |
| Male children 2-5 y | 0.16 ± 0.40 | 0.17 ± 0.43 | 0.24 ± 0.49 |
| Female children 2-5 y | 0.15 ± 0.40 | 0.15 ± 0.39 | 0.18 ± 0.44 |
| Male children 6-12 y | 0.35 ± 0.62 | 0.35 ± 0.63 | 0.35 ± 0.62 |
| Female children 6-12 y | 0.34 ± 0.60 | 0.33 ± 0.60 | 0.33 ± 0.61 |
| Male children 13-18 y | 0.45 ± 0.70 | 0.45 ± 0.70 | 0.45 ± 0.71 |
| Female children 13-18 y | 0.48 ± 0.75 | 0.47 ± 0.72 | 0.50 ± 0.75 |
| Male adults | 1.63 ± 1.03 | 1.77 ± 1.10 | 1.87 ± 1.20 |
| Female adults | 1.79 ± 1.05 | 1.94 ± 1.16 | 2.05 ± 1.23 |
| Geographic region, % |  |  |  |
| Central North | 19.8 | 20 | 19.9 |
| Central South | 14.3 | 14.1 | 14 |
| Mexico City | 27.4 | 27 | 27.2 |
| Northeast | 19.3 | 19.3 | 19.3 |
| Northwest | 10.2 | 10.3 | 10.4 |
| South | 9.1 | 9.3 | 9.3 |
| Unemployment, rate (mean ± SD) | 5.25 ± 1.33 | 5.18 ± 1.20 | 5.16 ± 1.35 |
| Minimum salary adjusted by consumer price index^2^, MX peso/day (mean ± SD) | 59.80 ± 1.51 | 60.31 ± 1.83 | 60.27 ± 1.85 |
| ^1^Estimates reflect mean or percentage of household-months.  ^2^Two-zone annual minimum salaries were adjusted by state-quarter consumer price index; the minimum salary of Mexico City for the1^st^ quarter in 2012 was used as the base.  Source: Authors’ own analyses and calculations based on data from Nielsen through its Mexico Consumer Panel Service (CPS) for the food and beverage categories for January 2012 – December 2014. | | | |
