# Supplementary material for: First-Year Evaluation of Mexico’s Tax on Nonessential Energy-Dense Foods: An Observational Study
Source: PLoS Med. 2016 Jul 5;13(7):e1002057. doi: 10.1371/journal.pmed.1002057 (PMC4933356; doi:10.1371/journal.pmed.1002057)
Supplement: S2 Table — (DOCX) [file pmed.1002057.s007.docx]

| **S2 Table.** Examples of food items for each food subcategory, and details on food classification process. | |
| --- | --- |
| **Food subcategory** | **Examples of food ítems** |
| Taxed |  |
| Salty snacks | Potato chips, corn chips, flour chips, fried pork skin, ready-to-eat popcorn, microwave popcorn, crackers, peanuts and seeds |
| Cereal based sweets | Cookies, pre-packaged sweet bread and cakes, cereal bars |
| Ready-to-eat cereals | Pre-prepared, ready-to-eat cereals |
| Non-cereal based sweets | Ice-cream, ice-pops, sorbets, “dulce de leche” or “cajeta”, fruit preserves, jellies, jam, milk modifiers with chocolate or other flavors |
| Untaxed |  |
| Sugar & sugar substitutes | Sugar, sugar substiturtes or non-caloric sweeteners |
| Cereals | Unsweetened breads and rolls, ready-to-eat cereals, pasta, corn, rice, oats |
| Dairy | Cheese, solid yogurt, cream, evaporated milk |
| Processed fruits & vegetables | Canned vegetables, tomato puree, frozen fruit |
| Salty snacks^1^ | Crackers, flour chips, fried pork skin |
| Non-cereal based sweets^1^ | Ice cream, sorbets, popsicles, “dulce de leche” or “cajeta”, fruit preserves, jellies, jam, gelatin, flan, pudding |
| Other | Soups, broth, baby food, powder coffee cream, chile sauces, catsup, condiments, salad dressing, mayonnaise, salt, canned tuna |
| **Food classification process** | |
| For a product to be taxed it has to comply with two conditions: be defined as “non-essential food” and have ≥275 kcal/100 g. A team of Mexican registered dieticians reviewed each product and assigned it into a tax/food category. Foods were classified as “taxed” or “untaxed” following the law definition of non-essential foods and by searching their energy content online. When it was unclear as to whether a food was considered non-essential, we contacted the Ministry of Finances, which is in charge of the tax administration. In some cases food producers also had similar questions and an official clarification was made in the Ministry of Finances website; in other cases we only obtained an unofficial interpretation from the Ministry of Finances. When the needed information to classify a product was not available online, our team conducted in-store checks. All products were classified into a “taxed” or “untaxed” category. Items that were at some point unclear represent 2.3% of total products purchased in 2014.  Examples of “non-essential” food definition clarifications: -Crackers with sugar content are only considered non-essential if they have <1200 mg of Na /100 g of product. -“Tostadas” (fried corn tortilla) and “totopos” (corn tortilla chips) are only considered non-essential if salt, chili pepper, spices or condiments are added after the corn flour has been baked, fried or dehydrated.  -Cereal based products with sugar are non-essential only if ready-to-eat (e.g., flours to prepare cake are essential) -Salty snacks are non-essential even if they are not ready-to-eat (e.g., microwave pop-corn is non-essential). | |
| ^1^Considered non-essential but with energy density <275 kcal/100 g; except crackers which were not considered non-essential if sodium content was >1200 mg/100 g. | |
