# Supplementary material for: First-Year Evaluation of Mexico’s Tax on Nonessential Energy-Dense Foods: An Observational Study
Source: PLoS Med. 2016 Jul 5;13(7):e1002057. doi: 10.1371/journal.pmed.1002057 (PMC4933356; doi:10.1371/journal.pmed.1002057)
Supplement: S3 Table — (DOCX) [file pmed.1002057.s008.docx]

| **S3 Table.** Main regression coefficients of the models used to predict volume purchased. | | | | |
| --- | --- | --- | --- | --- |
|  | **T**  **Post-tax period**  **(0=2012-2013;**  **1=2014)** | **S**  **2nd semester**  **(0=Jan-Jun;**  **1=Jul-Dec)** | **T*S**  **Post-tax and**  **semester interaction** | **Y**  **Year**  **(0=2012; 1=2013; 2=2014)** |
|  | *β (95% Confidence Interval)* | | | |
| **Taxed foods** |  |  |  |  |
| All sample | -16 (-30, -3) | 14 (7, 20) | -17 (-28, -6) | -8 (-17, 2) |
| Low SES | -38 (-66, -10) | 5 (-7, 16) | -12 (-29, 5) | 2 (-19, 23) |
| Medium SES | -19 (-37, -2) | 20 (10, 30) | -18 (-34, -2) | 1 (-13, 14) |
| High SES | -4 (-31, 23) | 8 (-6, 23) | -18 (-40, 4) | -24 (-44, -4) |
| **Untaxed foods** |  |  |  |  |
| All sample | 6 (-27, 40) | 25 (7, 43) | -23 (-49, 4) | 7 (-15, 30) |
| Low SES | -3 (-63, 57) | 15 (-15, 45) | -35 (-81, 11) | 35 (-11, 81) |
| Medium SES | -24 (-73, 26) | 30 (5, 55) | -25 (-63, 14) | 34 (2, 67) |
| High SES | 44 (-32, 120) | 31 (-8, 69) | -6 (-63, 51) | -49 (-107, 8) |
|  |  |  |  |  |
| **Taxed food subcategories** |  |  |  |  |
| **Probit model** |  |  |  |  |
| Salty snacks | -0.15 (-0.19, -0.1) | -0.02 (-0.04, 0.01) | -0.03 (-0.07, 0.01) | 0.07 (0.04, 0.1) |
| Cereal based sweets | -0.06 (-0.11, 0) | 0.06 (0.03, 0.09) | -0.09 (-0.13, -0.05) | 0.04 (0, 0.07) |
| RTE cereals | -0.05 (-0.09, -0.01) | 0.05 (0.03, 0.08) | -0.02 (-0.05, 0.02) | 0.01 (-0.02, 0.03) |
| Non-cereal based sweets | -0.07 (-0.11, -0.02) | -0.01 (-0.03, 0.02) | 0 (-0.04, 0.03) | 0.05 (0.03, 0.08) |
| **Linear model** |  |  |  |  |
| Salty snacks | 2 (-3, 8) | 4 (1, 7) | -4 (-8, 1) | -1 (-5, 3) |
| Cereal based sweets | 1 (-9, 10) | 17 (12, 22) | -14 (-22, -7) | -5 (-11, 2) |
| RTE cereals | 2 (-8, 12) | -1 (-6, 3) | 7 (0, 14) | -7 (-13, -1) |
| Non-cereal based sweets | 2 (-5, 9) | 2 (-1, 6) | 7 (0, 14) | 3 (-1, 7) |
| **Untaxed food subcategories** |  |  |  |  |
| **Probit model** |  |  |  |  |
| Sugar & sugar substitutes | -0.05 (-0.09, 0) | 0.07 (0.04, 0.09) | -0.03 (-0.06, 0.01) | 0.1 (0.07, 0.13) |
| Cereals | 0.04 (-0.03, 0.1) | 0.08 (0.05, 0.12) | -0.08 (-0.13, -0.03) | 0.02 (-0.02, 0.06) |
| Dairy | 0.01 (-0.07, 0.08) | 0.02 (-0.02, 0.06) | -0.11 (-0.17, -0.05) | 0.04 (-0.01, 0.08) |
| Processed fruits & vegetables | 0.04 (0, 0.08) | 0.1 (0.08, 0.12) | -0.04 (-0.07, -0.01) | 0 (-0.03, 0.02) |
| Salty snacks | -0.03 (-0.07, 0.02) | -0.06 (-0.09, -0.04) | -0.04 (-0.08, -0.01) | 0.02 (0, 0.05) |
| Non-cereal based sweets | 0.01 (-0.03, 0.05) | -0.01 (-0.04, 0.01) | -0.01 (-0.05, 0.03) | 0 (-0.02, 0.03) |
| Other | 0.02 (-0.03, 0.08) | -0.03 (-0.05, 0) | 0 (-0.04, 0.05) | -0.03 (-0.06, 0) |
| **Linear model** |  |  |  |  |
| Sugar & sugar substitutes | -13 (-47, 22) | 9 (-9, 27) | -5 (-36, 25) | 27 (6, 48) |
| Cereals | -5 (-18, 8) | 24 (18, 31) | 2 (-8, 12) | 3 (-6, 12) |
| Dairy | 14 (-5, 33) | 13 (3, 22) | -11 (-25, 3) | -13 (-26, -1) |
| Processed fruits & vegetables | 4 (-11, 18) | 18 (12, 24) | -3 (-12, 6) | 1 (-8, 10) |
| Salty snacks | 8 (3, 12) | -3 (-6, -1) | -5 (-8, -1) | -3 (-6, 0) |
| Non-cereal based sweets | 12 (-5, 29) | 1 (-7, 8) | 4 (-7, 15) | -9 (-18, 1) |
| Other | 15 (5, 25) | -2 (-7, 4) | 11 (3, 19) | -5 (-11, 2) |
| Source: Authors’ own analyses and calculations based on data from Nielsen through its Mexico Consumer Panel Service (CPS) for the food and beverage categories for January 2012 – December 2014. | | | | |
