# Supplementary material for: First-Year Evaluation of Mexico’s Tax on Nonessential Energy-Dense Foods: An Observational Study
Source: PLoS Med. 2016 Jul 5;13(7):e1002057. doi: 10.1371/journal.pmed.1002057 (PMC4933356; doi:10.1371/journal.pmed.1002057)
Supplement: S4 Table — (DOCX) [file pmed.1002057.s009.docx]

| **S4 Table.** National sales of mainly taxed foods in 2014 and percent change in sales from previous years reported by INEGI^1^ and Euromonitor^2^ | | | | | | |
| --- | --- | --- | --- | --- | --- | --- |
|  | **2014**  **g/capita/month** |  | **% change from previous year** | | | |
|  |  |  | **2011** | **2012** | **2013** | **2014** |
| **INEGI** |  |  |  |  |  |  |
| **Salty snacks** | **183** |  | **6.5** | **7.8** | **-0.5** | **-5.0** |
| Potato/corn/wheat chips & extruted snacks | 153 |  | 5.2 | 11.6 | -0.3 | -6.1 |
| Peanuts ("japanese" and spicy) | 24 |  | 4.0 | -7.5 | -1.9 | 1.6 |
| Popcorn | 5 |  | 41.8 | -38.4 | -0.1 | -3.8 |
| **Cereal based sweets** | **783** |  | **2.3** | **3.4** | **-0.8** | **-4.6** |
| Packaged/industrial pastries | 137 |  | -1.2 | 8.4 | 1.5 | -7.7 |
| Packaged/industrial cakes | 106 |  | 0.4 | 1.8 | -4.1 | -2.4 |
| Sweet biscuits | 539 |  | 3.5 | 2.4 | -0.8 | -4.3 |
| **Non-cereal based sweets** | **374** |  | **-1.6** | **-4.0** | **-6.2** | **-2.9** |
| Chocolates | 94 |  | -6.0 | -25.3 | -6.5 | -2.1 |
| Candies and gum | 246 |  | -0.2 | 1.3 | -7.4 | -1.3 |
| Pudins and milk based desserts | 34 |  | 4.7 | 15.6 | 1.6 | -16.4 |
| **TOTAL** | **1340** |  | **1.7** | **1.8** | **-2.3** | **-4.2** |
| **EUROMONITOR** |  |  |  |  |  |  |
| **Salty snacks** | **286** |  | **1.4** | **2.5** | **0.5** | **1.5** |
| Potato/corn/wheat chips & extruded snacks | 202 |  | 1.6 | 1.8 | 0.4 | 1.9 |
| Nuts | 43 |  | 0.1 | 6.1 | 1.4 | 1.2 |
| Popcorn | 41 |  | 1.9 | 2.0 | -0.1 | 0.1 |
| **Cereal based sweets** | **1,009** |  | **3.6** | **0.3** | **-0.2** | **-8.3** |
| Packaged/industrial pastries | 230 |  | 7.0 | 0.4 | 1.7 | -12.0 |
| Unpackaged/artisanal pastries | 286 |  | 5.5 | -0.1 | -0.2 | -13.3 |
| Packaged/industrial cakes | 79 |  | 0.2 | 0.1 | -5.6 | -9.0 |
| Unpackaged/artisanal cakes | 27 |  | 0.4 | 0.4 | 0.1 | -4.3 |
| Sweet biscuits | 361 |  | 0.8 | 0.5 | -0.7 | -2.9 |
| Cereal bars | 26 |  | 4.6 | 2.1 | 3.6 | 1.4 |
| **RTE cereals** | **71** |  | **1.2** | **0.6** | **-2.8** | **-8.2** |
| **Non-cereal based sweets** | **228** |  | **1.5** | **-4.9** | **-3.0** | **-6.2** |
| Chocolates | 45 |  | 8.8 | 7.1 | 6.1 | 3.0 |
| Candies and gum | 183 |  | 0.3 | -7.2 | -5.0 | -8.5 |
| **TOTAL** | **1,594** |  | **2.8** | **-0.1** | **-0.6** | **-6.3** |
| ^1^From the National Institute of Statistics, Geography and Informatics (INEGI) monthly manufacturer’s industry survey. Sales include exports. Data extracted from: <http://www.inegi.org.mx/sistemas/bie/> on September 2015  ^2^From Euromonitor International’s Passport Global Market. Sales presented are off-trade (from retail locations, such as supermarkets and convenient stores; does not include food services). Data extracted from <http://www.portal.euromonitor.com> via the UNC-Chapel Hill Libraries on September 2015. | | | | | | |
