# Supplementary material for: First-Year Evaluation of Mexico’s Tax on Nonessential Energy-Dense Foods: An Observational Study
Source: PLoS Med. 2016 Jul 5;13(7):e1002057. doi: 10.1371/journal.pmed.1002057 (PMC4933356; doi:10.1371/journal.pmed.1002057)
Supplement: S5 Table — (DOCX) [file pmed.1002057.s010.docx]

| **S5 Table.** National sales of mainly untaxed foods in 2014 and percent change in sales from previous years reported by INEGI^1^ and Euromonitor^2^ | | | | | | |
| --- | --- | --- | --- | --- | --- | --- |
|  | **2014**  **g/capita/month** |  | **% change from previous year** | | | |
|  |  |  | **2011** | **2012** | **2013** | **2014** |
| **INEGI** |  |  |  |  |  |  |
| Industrial bread | 350 |  | 0.8 | -0.5 | -1.8 | 1.7 |
| Pasta | 242 |  | 0.9 | 2.7 | -4.0 | 0.9 |
| Corn flour | 1,638 |  | 4.8 | -2.8 | -4.5 | 0.4 |
| Cheese | 262 |  | 4.7 | 4.0 | 5.0 | 15.3 |
| Vegetable oils | 802 |  | 11.3 | 3.3 | -5.9 | 2.4 |
| Butter and margarine | 44 |  | 7.0 | 28.6 | -8.5 | 8.0 |
| **TOTAL** | **3,338** |  | **5.7** | **0.2** | **-3.9** | **2.3** |
| **EUROMONITOR** |  |  |  |  |  |  |
| Packaged/Industrial Bread | 404 |  | -0.7 | 1.6 | -2.0 | -0.8 |
| Unpackaged/Artisanal Bread | 7,347 |  | -5.3 | -0.8 | -0.2 | 0.5 |
| Pasta | 228 |  | 1.6 | 1.0 | 0.8 | 1.1 |
| Rice | 420 |  | 0.5 | 0.7 | 0.6 | 0.7 |
| Cheese | 343 |  | 0.7 | 1.2 | 3.5 | 1.9 |
| Canned/Preserved Food | 396 |  | -0.5 | 0.9 | -2.2 | 3.4 |
| Sauces, Dressings and Condiments | 654 |  | 1.8 | 0.2 | 3.1 | 1.4 |
| Soups | 19 |  | -0.2 | -11.6 | 1.8 | 0.0 |
| Vegetable oils | 571 |  | 1.3 | 1.1 | 1.0 | 1.4 |
| Butter and margarine | 26 |  | -0.1 | -1.5 | 0.0 | 0.5 |
| Fruits, vegetables and starchy roots | 8,396 |  | -5.6 | -0.5 | 0.7 | 0.3 |
| Meat, eggs, fish and seafood | 4,012 |  | -1.5 | 1.8 | 0.5 | 0.5 |
| Legumes | 478 |  | -64.1 | 32.4 | -0.1 | -0.6 |
| **TOTAL** | **23,295** |  | **-4.8** | **0.7** | **0.4** | **0.5** |
| ^1^From the National Institute of Statistics, Geography and Informatics (INEGI) monthly manufacturer’s industry survey. Sales include exports. Data extracted from: <http://www.inegi.org.mx/sistemas/bie/> on September 2015.  ^2^From Euromonitor International’s Passport Global Market. Sales presented are off-trade (from retail locations, such as supermarkets and convenient stores; does not include food services). Data extracted from <http://www.portal.euromonitor.com> via the UNC-Chapel Hill Libraries on September 2015. | | | | | | |
